# Elevated Anti-Mycoplasma pneumoniae IgG Titers in Chronic Epipharyngitis: Insights Into Persistent Immune Dysregulation

**DOI:** 10.7759/cureus.115653

**Published:** 2026-09-02

**Authors:** Manabu Mogitate, Sachie Matsuda, Kazuhiro Matsuda

**Affiliations:** 1 Otolaryngology, Mogitate ENT Clinic, Kawasaki, JPN; 2 Dermatology, Shimbashi Dermatology Clinic, Tokyo, JPN; 3 Mycoplasma Infectious Diseases Research Center, M Bio Technology Inc, Tokyo, JPN

**Keywords:** chronic epipharyngitis, chronic inflammation, epipharyngeal abrasive therapy, immunoglobulin g, mucosal immunity, mycoplasma pneumoniae, persistent immune dysregulation, systemic symptoms

## Abstract

Objective

Chronic epipharyngitis is increasingly recognized as a condition that may involve not only local mucosal inflammation but also altered immune responses and diverse systemic symptoms in some patients. This study aimed to investigate the clinical significance of elevated anti-*Mycoplasma pneumoniae* IgG titers in patients with chronic epipharyngitis and to explore their potential relationship with persistent immune dysregulation and systemic symptoms.

Methods

This retrospective observational study included 288 patients with chronic epipharyngitis and nine healthy controls. Serum antibodies against *Mycoplasma pneumoniae* and *Mycoplasma fermentans* lipid antigens were measured before treatment. Endoscopic inflammatory scores were evaluated longitudinally before and after epipharyngeal abrasive therapy (EAT). Associations between baseline antibody levels and clinical outcomes were analyzed.

Results

Baseline anti-*Mycoplasma pneumoniae* IgG levels demonstrated substantial interindividual variability among patients with chronic epipharyngitis. Patients with higher baseline IgG levels exhibited significantly greater improvement in endoscopic inflammatory scores following EAT and showed higher responder rates than those with lower baseline levels. In contrast, antibody levels were not significantly different between patients and healthy controls. These findings suggest that elevated anti-*Mycoplasma pneumoniae* IgG titers may identify a subgroup of patients with chronic epipharyngitis characterized by enhanced mucosal immune responsiveness and distinct immunological features.

Conclusions

Elevated anti-*Mycoplasma pneumoniae* IgG titers may reflect persistent antigenic imprinting and a distinct host immune background rather than active infection in patients with chronic epipharyngitis. The findings provide insight into a potentially distinct immunological subgroup and may contribute to understanding systemic manifestations frequently observed in chronic epipharyngitis, including fatigue, cognitive dysfunction, and autonomic symptoms. Further studies are warranted to clarify the relationship between anti-*Mycoplasma pneumoniae* immunity, chronic epipharyngeal inflammation, and systemic symptoms.

## Introduction

Chronic epipharyngitis is a chronic inflammatory disorder of the epipharyngeal mucosa and has traditionally been regarded as a localized disease of the upper airway. However, accumulating evidence suggests that chronic epipharyngitis is associated not only with local symptoms such as postnasal drip, throat discomfort, and chronic cough but also with diverse systemic manifestations, including chronic fatigue, cognitive dysfunction (brain fog), autonomic symptoms, and various immune-mediated disorders. Recent studies have therefore proposed that chronic epipharyngitis should be viewed as a condition involving persistent immune dysregulation rather than a simple local inflammatory process [1-4]. However, these systemic manifestations and immunological mechanisms were not directly evaluated in the present study and should be regarded as background concepts derived from prior literature rather than established features of this cohort.

The epipharynx is anatomically positioned at the intersection of the respiratory tract and mucosal immune system and contains abundant lymphoid tissue known as nasopharynx-associated lymphoid tissue. As a component of the mucosal immune network, the epipharynx is continuously exposed to inhaled microorganisms, environmental antigens, and airborne particles. Chronic inflammatory stimulation at this site may induce prolonged immune activation and contribute to systemic inflammatory responses beyond the local mucosa. Recent studies have demonstrated increased expression of inflammatory cytokines, including interleukin-6 (IL-6) and tumor necrosis factor-alpha (TNF-α), in patients with chronic epipharyngitis, supporting the concept of persistent mucosal immune activation [5-7].

Growing attention has recently focused on the relationship between chronic inflammation and persistent antigenic stimulation. In myalgic encephalomyelitis/chronic fatigue syndrome (ME/CFS), long COVID, and other post-infectious disorders, pathological symptoms may persist even after resolution of acute infection. These conditions are increasingly considered to represent a state of chronic immune dysregulation induced by prior antigen exposure and immune imprinting rather than ongoing pathogen replication [8-10]. Similar mechanisms may contribute to the pathophysiology of chronic epipharyngitis, in which persistent immune activation remains detectable despite the absence of clinically apparent acute infection.

Among potential sources of persistent antigenic stimulation, *Mycoplasma pneumoniae* has attracted considerable interest. Although acute *M. pneumoniae* infection is primarily recognized as a respiratory infection, several studies have suggested that mycoplasma-derived antigens may induce prolonged immune responses that persist after the acute phase. In particular, species-specific lipid antigens of *M. pneumoniae* have been shown to elicit durable humoral immune responses and may serve as indicators of cumulative antigen exposure rather than active infection [11-14]. Persistent elevation of anti-*Mycoplasma pneumoniae* immunoglobulin G (IgG) titers may therefore reflect a distinct immunological state characterized by ongoing immune activation and altered inflammatory responsiveness.

Epipharyngeal abrasive therapy (EAT) is widely used in Japan for the treatment of chronic epipharyngitis and has been reported to improve both local inflammatory findings and systemic symptoms. Previous studies have demonstrated that EAT reduces inflammatory cytokine expression, decreases exhaled nitric oxide levels, modulates local immune cell populations, and improves symptoms in patients with chronic fatigue syndrome and long COVID. Furthermore, recent work has suggested that EAT may influence neuroimmune interactions through autonomic nervous system modulation [15-21]. These observations support the concept that chronic epipharyngitis represents a disorder involving complex interactions among the immune, nervous, and inflammatory systems.

We hypothesized that elevated anti-*Mycoplasma pneumoniae* IgG titers may identify a subgroup of patients with chronic epipharyngitis characterized by persistent immune dysregulation and associated systemic symptoms. Therefore, the present study investigated serum anti-*Mycoplasma pneumoniae* IgG titers in patients with chronic epipharyngitis and explored their potential clinical significance as a biomarker of persistent immune dysregulation and systemic symptomatology.

## Materials and methods

Study design and participants

This retrospective observational study was conducted at Mogitate ENT Clinic (Kawasaki, Japan). Consecutive patients diagnosed with chronic epipharyngitis who underwent serum testing for anti-*Mycoplasma pneumoniae* lipid-antigen antibodies between June 2016 and May 2017 were included. Chronic epipharyngitis was diagnosed based on persistent epipharyngeal symptoms, including postnasal drip, throat discomfort, chronic cough, and pharyngeal irritation, together with characteristic endoscopic findings and the presence of contact bleeding during epipharyngeal abrasion. Patients were eligible if baseline serological data and longitudinal endoscopic evaluations were available, whereas those with acute upper respiratory infection, malignancy, systemic immunosuppressive therapy, or incomplete clinical records were excluded. For the PIgG-stratified and responder analyses, only patients with complete longitudinal endoscopic data at all required timepoints (EAT0, EAT12, and EAT24) were included (n = 87). The remaining 201 patients were excluded solely due to incomplete follow-up data, not based on PIgG levels or clinical characteristics. A total of 288 patients with chronic epipharyngitis and nine healthy volunteers without evidence of chronic epipharyngitis were included in the analysis. The healthy controls were enrolled to provide baseline reference values for anti-*Mycoplasma pneumoniae* IgG titers and to facilitate comparison of immune-related findings between healthy individuals and patients with chronic epipharyngitis.

Endoscopic evaluation

Endoscopic examination was performed using a flexible nasopharyngoscope (EPK-i7000 processor and VNL-1190STK endoscope; Pentax Medical, Tokyo, Japan). The severity of epipharyngeal inflammation was evaluated using a standardized endoscopic scoring system established by the Epipharyngeal Abrasive Therapy (EAT) Review Committee. Mucosal redness, swelling, mucus adhesion, and contact bleeding were assessed during each examination. Contact bleeding was defined as spontaneous mucosal bleeding elicited by gentle swabbing with standardized minimal pressure prior to any abrasive motion. Bleeding that occurred only after deliberate abrasion or increased pressure was not classified as contact bleeding, ensuring that contact bleeding reflected mucosal fragility rather than operator-induced trauma. Endoscopic scores were recorded before treatment (EAT0), after 12 treatment sessions (EAT12), and after 24 treatment sessions (EAT24). Improvement in endoscopic scores was used as an objective indicator of mucosal recovery and treatment responsiveness.

Epipharyngeal abrasive therapy

EAT was performed once weekly according to standard clinical practice. A cotton applicator soaked in zinc chloride solution was applied transnasally and transorally to the epipharyngeal mucosa under endoscopic guidance. The zinc chloride solution used for EAT had a concentration of 0.5%, and approximately 0.3-0.5 mL was applied during each session. Mucosal contact was maintained for approximately 5-10 seconds. All procedures followed a standardized technique to minimize operator variability across patients. Mechanical abrasion was used to remove inflammatory exudates and stimulate healing of the inflamed mucosa. Contact bleeding during abrasion was recorded as a characteristic finding of chronic epipharyngitis. Serial endoscopic examinations were performed throughout treatment to evaluate mucosal recovery.

Sample collection

Peripheral blood samples were collected before initiation of treatment. In healthy controls, blood samples were obtained at the time of study enrollment. Serum was prepared according to standard laboratory procedures and stored until analysis.

Laboratory measurements

Serum antibodies directed against species-specific lipid antigens of *Mycoplasma pneumoniae* and* Mycoplasma fermentans* were measured using validated lipid-antigen serological assays. Anti-Mycoplasma pneumoniae lipid-antigen IgG was measured using a commercially available ELISA (Matsuda Laboratory, Tokyo, Japan). Results were reported in units (U/mL) according to the manufacturer’s calibration curve. The reference range was 0.00-0.29 U/mL, and values ≥0.30 U/mL were considered elevated. Assay calibration and quality control procedures followed standard laboratory protocols. The biomarkers analyzed included anti-*Mycoplasma pneumoniae* lipid-antigen IgG (PIgG), anti-*Mycoplasma pneumoniae* lipid-antigen IgM (PIgM), anti-*Mycoplasma fermentans* lipid-antigen IgG (FIgG), and anti-*Mycoplasma fermentans* lipid-antigen IgM (FIgM). Particular attention was paid to PIgG because elevated IgG titers were hypothesized to reflect persistent antigenic imprinting and immune dysregulation rather than active infection. Patients were categorized into three groups based on baseline PIgG levels (<0.300, 0.300-0.499, ≥0.500). These thresholds were determined empirically according to distribution patterns consistently observed in clinical practice, as described by Matsuda et al. In routine epipharyngeal immunology, PIgG values ≥0.500 have been associated with stronger mucosal inflammatory responses and greater therapeutic responsiveness, whereas values <0.300 typically correspond to minimal serological reactivity. The intermediate range (0.300-0.499) represents a transitional zone without a clearly defined clinical phenotype. Because no validated PIgG reference ranges or standardized cutoffs currently exist for chronic epipharyngitis, this experience-based stratification was adopted for the present study.

Statistical analysis

Continuous variables were expressed as mean ± standard deviation (SD) or median with interquartile range (IQR), as appropriate. Comparisons between patients and healthy controls were performed using the Mann-Whitney U test. Longitudinal changes in endoscopic scores were evaluated using the Wilcoxon signed-rank test. Patients were stratified according to baseline PIgG levels, and differences in treatment response were analyzed using the Kruskal-Wallis test with post hoc multiple comparisons where appropriate. Correlations between variables were assessed using Spearman’s rank correlation coefficient. All statistical analyses were performed using Python version 3.10 with the NumPy, SciPy, and pandas libraries. A two-sided p-value < 0.05 was considered statistically significant.

Ethical approval

This study was conducted in accordance with the principles of the Declaration of Helsinki and was approved by the Joint Institutional Review Board (IRB No. 14000050; Approval No. 20170721-4482). Because this was a retrospective study using routinely collected clinical data, an opt-out consent procedure was adopted. Patients were informed of the study through public disclosure and were provided with the opportunity to decline participation. Only data from patients who did not opt out were included in the analysis.

## Results

Baseline characteristics

A total of 288 patients with chronic epipharyngitis and nine healthy controls were included in this study. The patient cohort consisted of 206 female patients (71.5%) and 82 male patients (28.5%), with a median age of 45 years. Baseline measurements of anti-*Mycoplasma pneumoniae* and anti-*Mycoplasma fermentans* lipid-antigen antibodies demonstrated substantial interindividual variability among patients. Baseline demographic and clinical characteristics are summarized in Table 1.

**Table 1:** Baseline characteristics of the study population (n = 288) Baseline demographic characteristics, including age, sex distribution, and baseline anti-*Mycoplasma *antibody profiles, are presented for patients with chronic epipharyngitis and healthy controls.

Variable	Value
Sex	
Female	206 (71.5%)
Male	82 (28.5%)
Age, years	
Median (IQR)	45 (35–53)
Laboratory findings	
PIgG	0.50 ± 0.65
PIgM	0.08 ± 0.20
FIgG	0.04 ± 0.17
FIgM	0.50 ± 0.55

Improvement of endoscopic findings following EAT

Repeated EAT resulted in significant improvement of endoscopic inflammatory findings. The median endoscopic score decreased progressively from baseline (EAT0) to EAT12 and EAT24, indicating substantial mucosal recovery during treatment. Improvements were statistically significant for all major comparisons (all p < 0.001). These findings confirmed the therapeutic effectiveness of EAT in patients with chronic epipharyngitis. Detailed longitudinal changes in endoscopic scores are shown in Figure 1A and summarized in Table 2. Endoscopic score changes stratified according to baseline PIgG status are shown in Figure 1B.

**Table 2:** Longitudinal changes in endoscopic scores following EAT Median endoscopic inflammatory scores at baseline (EAT0), after 12 treatment sessions (EAT12), and after 24 treatment sessions (EAT24). Longitudinal changes were evaluated using the Wilcoxon signed-rank test, and effect sizes are presented where applicable. EAT, epipharyngeal abrasive therapy

Time point/Comparison	Median (IQR)	p-value (Wilcoxon)	Effect size (r)
EAT0	4 (3–5)	–	–
EAT12	2 (2–3)	<0.001	0.65（large）
EAT24	1 (1–2)	<0.001	0.75（large）
EAT0 vs EAT12	–	<0.001	large
EAT12 vs EAT24	–	0.01	moderate
EAT0 vs EAT24	–	<0.001	

**Figure 1:**
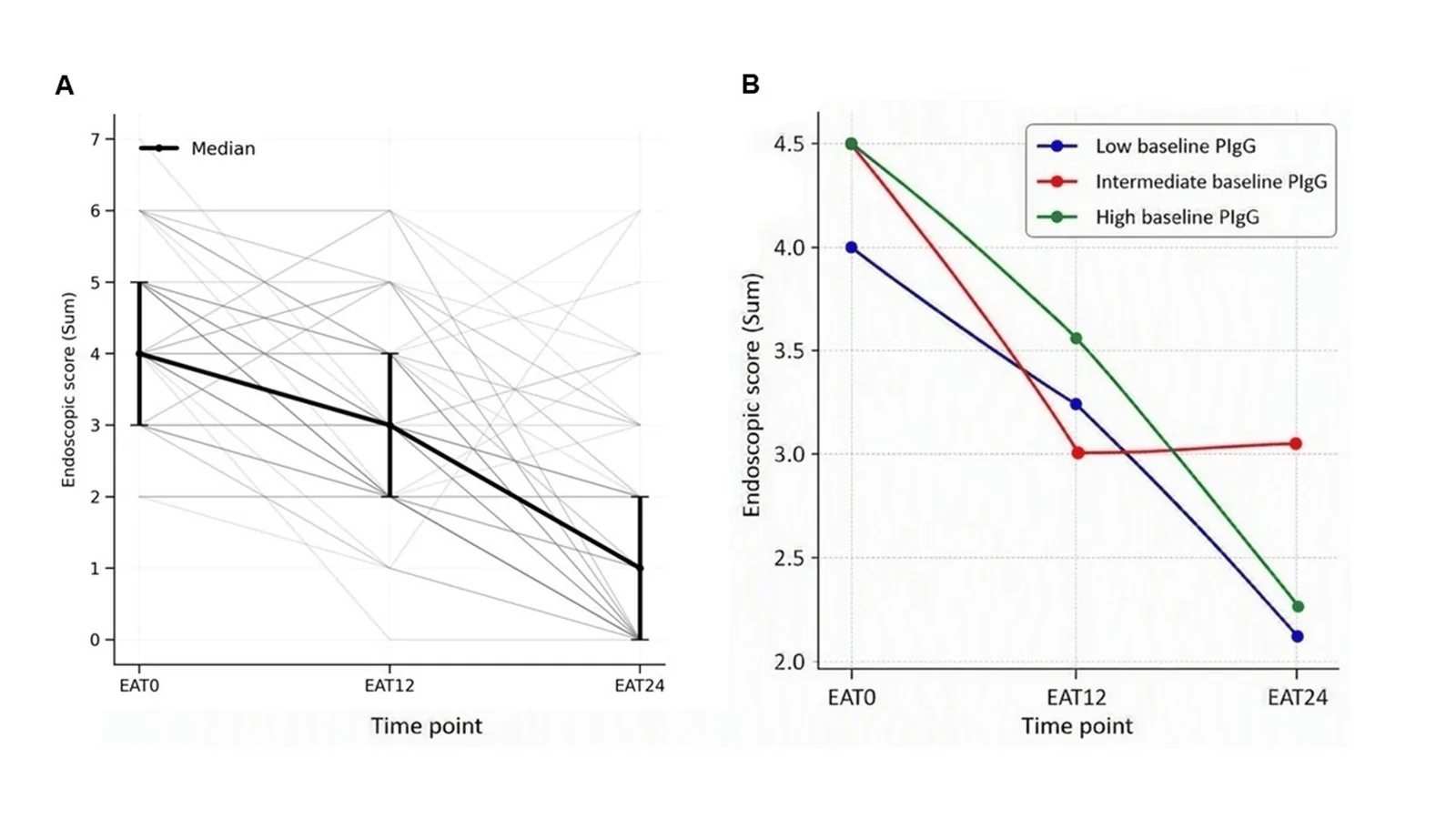
Longitudinal changes in endoscopic inflammatory scores following epipharyngeal abrasive therapy Changes in endoscopic inflammatory scores during epipharyngeal abrasive therapy (EAT). (A) Individual longitudinal trajectories and median endoscopic inflammatory scores at baseline (EAT0), after 12 treatment sessions (EAT12), and after 24 treatment sessions (EAT24). Progressive improvement in endoscopic findings was observed throughout treatment. (B) Median endoscopic inflammatory scores stratified according to baseline PIgG status. All groups demonstrated improvement over time, with greater reductions observed in patients with elevated baseline PIgG titers.

Comparison of baseline antibody profiles between patients and healthy controls

Baseline antibody levels against *Mycoplasma pneumoniae* and *Mycoplasma fermentans* lipid antigens were compared between patients and healthy controls.

No significant differences were observed for serum anti-*Mycoplasma pneumoniae* lipid-antigen IgG (Figure 2A), serum anti-*Mycoplasma pneumoniae* lipid-antigen IgM (Figure 2B), serum anti-*Mycoplasma fermentans* lipid-antigen IgG (Figure 2C), or serum anti-*Mycoplasma fermentans* lipid-antigen IgM (Figure 2D). Although antibody levels varied considerably among individual patients, the distributions largely overlapped with those of healthy controls. These findings suggest that anti-Mycoplasma lipid-antigen antibody levels are not diagnostic biomarkers of chronic epipharyngitis.

**Figure 2:**
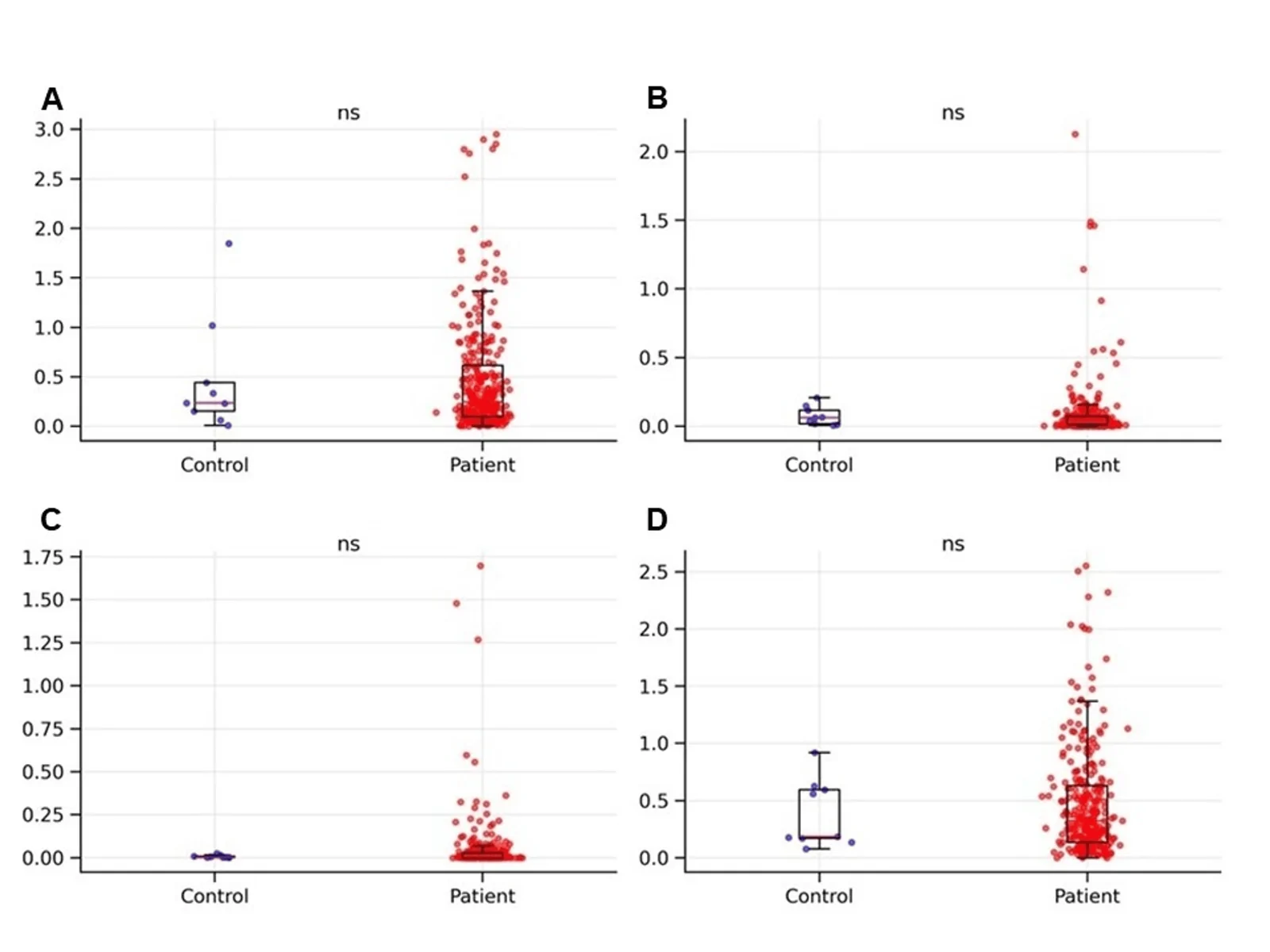
Baseline lipid-antigen antibody profiles in patients with chronic epipharyngitis and healthy controls (A) Serum anti-*Mycoplasma pneumoniae* lipid-antigen IgG (PIgG). (B) Serum anti-*Mycoplasma pneumoniae* lipid-antigen IgM (PIgM). (C) Serum anti-*Mycoplasma fermentans* lipid-antigen IgG (FIgG). (D) Serum anti-*Mycoplasma fermentans* lipid-antigen IgM (FIgM). No significant differences were observed between patients with chronic epipharyngitis and healthy controls for any antibody measurement, indicating that these serological markers are not diagnostic biomarkers of chronic epipharyngitis. ns, not significant; IgG, immunoglobulin G; IgM, immunoglobulin M.

Association between baseline PIgG levels and endoscopic improvement

Patients were stratified according to baseline PIgG levels and compared for treatment responsiveness.

The magnitude of endoscopic improvement differed significantly among PIgG-defined groups. Patients with high baseline PIgG levels demonstrated significantly greater reductions in endoscopic inflammatory scores than patients with low baseline PIgG levels (Kruskal-Wallis test, p = 0.0136). The greatest improvement was consistently observed in the high-PIgG subgroup.

These findings suggest that elevated PIgG levels are associated with enhanced mucosal responsiveness to EAT. The relationship between baseline PIgG levels and treatment responsiveness is illustrated in Figure 3.

**Figure 3:**
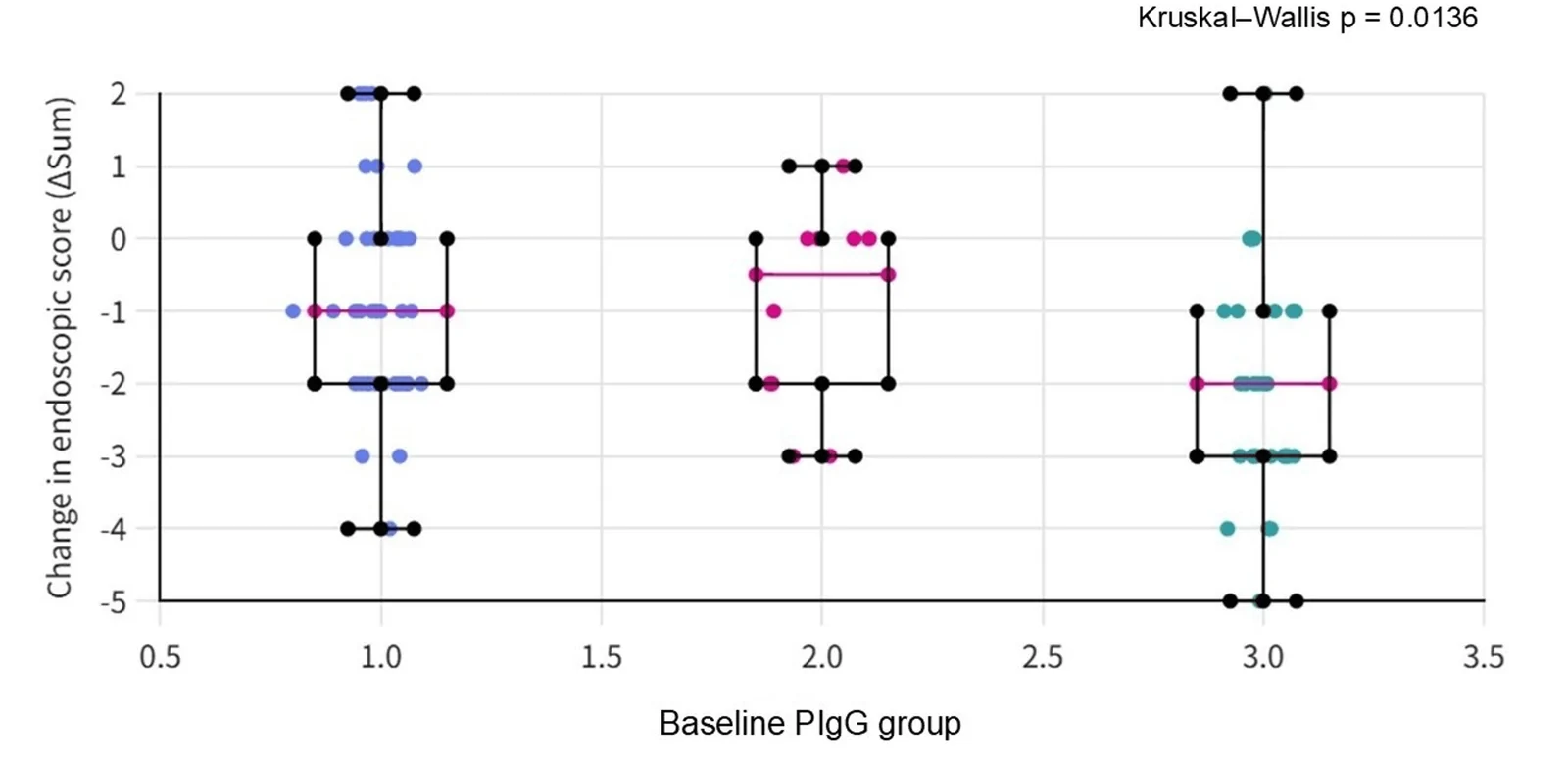
Association between the baseline anti-Mycoplasma pneumoniae lipid-antigen IgG level and mucosal responsiveness to therapy Changes in endoscopic inflammatory scores (ΔSum score) were compared among low-, intermediate-, and high-PIgG groups. Greater reductions in inflammatory scores were observed in patients with higher baseline PIgG levels, indicating enhanced mucosal recovery following epipharyngeal abrasive therapy.

Responder analysis

Responder rates were further evaluated according to baseline PIgG status. The high-PIgG group demonstrated the highest responder rate (28/33 (84.8%)), compared with 27/44 (61.4%) in the low-PIgG group and 6/10 (60.0%) in the intermediate-PIgG group. The difference between the high- and low-PIgG groups was statistically significant (p = 0.03). These results indicate that patients with elevated baseline PIgG levels were more likely to achieve clinically meaningful improvement following EAT. Responder rates according to the baseline PIgG status are shown in Figure 4.

**Figure 4:**
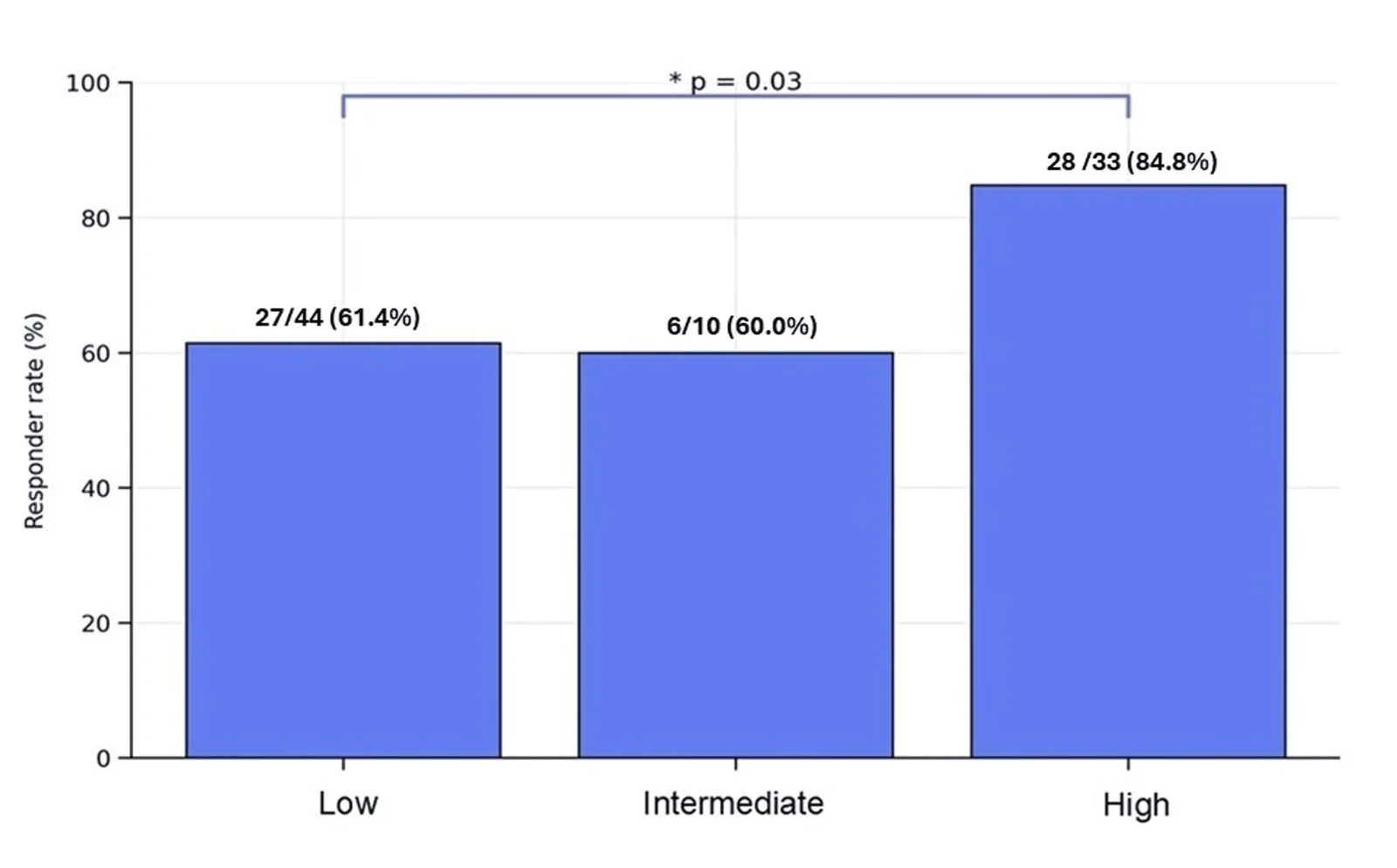
Treatment responsiveness according to the baseline anti-Mycoplasma pneumoniae lipid-antigen IgG status Patients were stratified into low, intermediate, and high baseline PIgG groups. Responder rates were 27/44 (61.4%) in the low-PIgG group, 6/10 (60.0%) in the intermediate-PIgG group, and 28/33 (84.8%) in the high-PIgG group. The responder rate was significantly higher in the high-PIgG group than in the low-PIgG group (p = 0.03). These findings suggest that elevated baseline PIgG levels are associated with enhanced responsiveness to epipharyngeal abrasive therapy (EAT). PIgG, anti-*Mycoplasma pneumoniae* lipid-antigen immunoglobulin G.

Dissociation between antibody levels and clinical improvement

Despite substantial improvement in endoscopic inflammatory findings during treatment, changes in antibody levels did not parallel clinical recovery.

Baseline PIgG levels were associated with subsequent treatment responsiveness; however, longitudinal antibody dynamics were not correlated with endoscopic improvement. This finding suggests that anti-*Mycoplasma pneumoniae* IgG reflects an underlying immunological state rather than ongoing inflammatory activity itself.

## Discussion

The present study suggests that elevated PIgG levels are not useful as diagnostic biomarkers of chronic epipharyngitis but may be associated with greater mucosal improvement during EAT. Patients with higher baseline PIgG levels demonstrated significantly greater improvement in endoscopic inflammatory findings and higher responder rates following treatment. These observations suggest that elevated PIgG may identify a distinct subgroup of patients with chronic epipharyngitis characterized by a unique immunological background rather than active infection.

Chronic epipharyngitis has traditionally been regarded as a localized inflammatory disease of the epipharyngeal mucosa. However, increasing evidence suggests that it is associated with systemic manifestations including fatigue, cognitive dysfunction, autonomic symptoms, and immune-mediated disorders [1-4]. Recent studies have demonstrated increased expression of inflammatory cytokines such as IL-6 and TNF-α in the epipharyngeal mucosa and have shown that EAT suppresses these inflammatory pathways [6]. In addition, increased numbers of epipharyngeal CD4-positive cells have been reported in patients with chronic epipharyngitis and were significantly reduced following EAT [7]. Collectively, these findings support the concept that chronic epipharyngitis represents a state of persistent mucosal immune activation rather than merely a local inflammatory disorder. Furthermore, chronic epipharyngitis has been implicated in systemic conditions such as chronic fatigue syndrome and IgA nephropathy, supporting the existence of an epipharynx-systemic disease axis [22,23]. However, the present study did not directly evaluate systemic symptoms or immunological mechanisms, and these concepts should be interpreted as background information derived from prior literature rather than findings demonstrated in this cohort.

A notable observation in the present study was the absence of significant differences in PIgG levels between patients and healthy controls. This finding suggests that elevated PIgG does not function as a disease-specific diagnostic marker. Instead, PIgG may reflect cumulative antigen exposure and long-term immunological memory. Previous investigations of Mycoplasma pneumoniae lipid antigens demonstrated that these glycolipid structures are species-specific immunodeterminants capable of inducing durable humoral immune responses [11,12]. Therefore, elevated PIgG levels may represent an immunological footprint of prior antigen exposure rather than evidence of active microbial infection.

Recent advances in immunology have highlighted the importance of immune imprinting, in which previous antigen exposure influences subsequent immune responses and inflammatory behavior [8]. Lipid antigens derived from microorganisms can be presented through CD1-mediated pathways and activate natural killer T cells and other lipid-reactive immune populations [13,14]. Consequently, persistent elevation of PIgG may indicate a host immune state characterized by altered responsiveness to inflammatory stimuli. From this perspective, our findings raise the possibility that patients with elevated PIgG represent an immunologically distinct subgroup with enhanced capacity for mucosal remodeling and recovery following EAT.

Another important finding was the dissociation between antibody status and disease activity. Although baseline PIgG levels were associated with subsequent treatment responsiveness, longitudinal clinical improvement did not parallel antibody dynamics. This observation suggests that circulating antibodies may serve as indicators of underlying immune status rather than direct markers of ongoing mucosal inflammation. Similar dissociations between systemic biomarkers and local tissue inflammation have been reported in various chronic inflammatory conditions [2,8]. The present findings support the concept that chronic epipharyngitis may represent a compartmentalized mucosal immune disorder in which local inflammatory activity and systemic immune markers are only partially correlated.

The relationship between chronic immune activation and systemic symptoms has attracted growing attention in recent years. Persistent immune dysregulation has been implicated in post-infectious conditions such as ME/CFS and long COVID, where fatigue, brain fog, sleep disturbance, and autonomic dysfunction persist despite the absence of active infection [9,10,17,18]. These conditions are increasingly understood as disorders of immune dysregulation and altered host responses rather than direct pathogen persistence. Additional observations linking epipharyngeal inflammation with neuroimmune dysfunction have also been reported, including hypotheses involving immune-neuroendocrine interactions in chronic fatigue syndrome [24]. Although the present study did not directly evaluate systemic symptoms, the association between elevated PIgG and enhanced mucosal immune responsiveness raises the possibility that similar immunological mechanisms may be operating in a subset of patients with chronic epipharyngitis.

Recent studies have further suggested that EAT influences not only local inflammation but also neuroimmune interactions through autonomic nervous system modulation [18-20]. Ito proposed that EAT may exert therapeutic effects through interactions among immune, autonomic, and endocrine systems within the framework of Psycho-Neuro-Endocrino-Immunology (PNEI) [4,19,20]. In addition, clinical studies have reported beneficial effects of EAT on long COVID, chronic cough, and autonomic dysfunction-related conditions, while molecular investigations have demonstrated the clearance of residual SARS-CoV-2-related signals and transcriptional changes within the epipharyngeal mucosa following treatment [17,25-27]. Although the present study was not designed to evaluate autonomic function or neuroimmune pathways directly, the identification of a PIgG-high subgroup may provide a useful framework for future investigations exploring relationships between immune dysregulation, autonomic symptoms, and systemic manifestations in chronic epipharyngitis.

The present study has several limitations. First, this was a retrospective single-center study. Second, the number of healthy controls was relatively small. Third, detailed immunological analyses including cytokine profiling, flow cytometry, and cellular phenotyping were not performed. Fourth, systemic symptoms such as fatigue, autonomic dysfunction, and cognitive impairment were not quantitatively assessed. Therefore, causal relationships between elevated PIgG, immune dysregulation, and systemic manifestations remain speculative. Future prospective studies incorporating immunological and clinical outcome measures are warranted.

## Conclusions

Elevated anti-*Mycoplasma pneumoniae* IgG titers were associated with greater mucosal responsiveness to EAT but not with disease diagnosis itself. These findings suggest that elevated PIgG may identify a distinct immunological subgroup of patients with chronic epipharyngitis who may exhibit altered mucosal inflammatory responsiveness rather than active infection. Further investigation is needed to clarify the relationship between PIgG, chronic mucosal inflammation, and systemic symptoms including fatigue, cognitive dysfunction, and autonomic complaints.
